# MicroRNA applications for prostate, ovarian and breast cancer in the era of precision medicine

**DOI:** 10.1530/ERC-16-0525

**Published:** 2017-03-13

**Authors:** Bethany Smith, Priyanka Agarwal, Neil A Bhowmick

**Affiliations:** 1 Department of MedicineSamuel Ochin Comprehensive Cancer Institute, Cedars-Sinai Medical Center, Los Angeles, California, USA; 2 Greater Los Angeles Veterans AdministrationLos Angeles, California, USA

**Keywords:** biomarker, TGF-β, clinical trials, microRNA, microenvironment

## Abstract

The high degree of conservation in microRNA from *Caenorhabditis* *elegans* to humans has enabled relatively rapid implementation of findings in model systems to the clinic. The convergence of the capacity for genomic screening being implemented in the prevailing precision medicine initiative and the capabilities of microRNA to address these changes holds significant promise. However, prostate, ovarian and breast cancers are heterogeneous and face issues of evolving therapeutic resistance. The transforming growth factor-beta (*TGFβ*) signaling axis plays an important role in the progression of these cancers by regulating microRNAs. Reciprocally, microRNAs regulate *TGFβ* actions during cancer progression. One must consider the expression of miRNA in the tumor microenvironment a source of biomarkers of disease progression and a viable target for therapeutic targeting. The differential expression pattern of microRNAs in health and disease, therapeutic response and resistance has resulted in its application as robust biomarkers. With two microRNA mimetics in ongoing restorative clinical trials, the paradigm for future clinical studies rests on the current observational trials to validate microRNA markers of disease progression. Some of today’s biomarkers can be translated to the next generation of microRNA-based therapies.

## Introduction

Precision medicine refers to leveraging larger scale somatic or genomic data in the informing medical decision-making. Although the principles of precision medicine are extending into proteomics and metabolomics, a vast amount of patient data currently exists in the form of genetic material. The genome expresses more non-coding RNA than the code for proteins. First recognized in 1993 in *Caenorhabditis elegans*, the noncoding RNAs, let-7 and lin-4 induced mRNA degradation (Lee *et al.* 1993). The mature miR let-7 sequence, conserved from *C. elegans* to humans, is downregulated in multiple cancers, which highlights the significance of the 3’UTR region during gene regulation (Pasquinelli 2000, Vella *et al.* 2004). MicroRNA (miR), most studied constituent of the non-coding RNAs, was termed to describe small (~22 nt) RNA regulators that influence gene functions (Lagos-Quintana *et al.* 2001). About 30–50% of all protein-coding genes are possibly regulated by miRs in health and deregulated in disease. However, the exquisite tissue and developmental stage specificity of miR expression mean that the regulation of miRs themselves can determine disease progression (Lagos-Quintana *et al.* 2001, Ruvkun 2001, Calin *et al.* 2002). Calin and coworkers found that specific miR downregulation was associated with the incidences of B cell chronic lymphocytic leukemia (Calin *et al.* 2002). Now what was once recognized as the passive circulation of miR (found in all body fluids) is emerging as signaling molecules between cells and robust biomarkers of cancer progression and therapy response.

Gene regulation by miRs and reciprocal regulation of miRs have now been studied for over 15 years and extensively reviewed (Vidigal & Ventura 2015). Briefly, miRs are transcribed as pri-miRNAs that are cleaved by the Drosha/DGCR8 complex in the nucleus (Han *et al.* 2004). The resulting hairpin-shaped pre-miRNA is further processed by Dicer in the cytoplasm (Zhang *et al.* 2002). The mature miRs employ the RNA-induced silencing complex (RISC) in the two mechanisms of gene regulation through mRNA degradation and translation inhibition (Fabian & Sonenberg 2012). The core of complex includes an argonaute protein for miR targeting and GW182 protein to serve as the scaffold on the mRNA poly-A tail (Song *et al.* 2004, Hutvagner & Simard 2008). The degree of pairing between the miR and the target mRNA seems to be a determinant of the mechanism of regulation. Direct complementarity will induce target mRNA cleavage by argonaute and be immediately repressive. In the indirect or imperfect complementarity scenario, miRs bind to the 3′ UTR prior to the poly-A tail to cause deadenylation of the mRNA. Loss of the poly-A tail leads to mRNA degradation. Imperfect complementation can additionally induce translational repression by blocking initiation of causing ribosomal drop-off. While the perfect pairing may be a more efficient repressor, there is a possibility that partial complementation may have a greater long-term efficacy: an issue considered in therapeutic design. Although miRs can bind the ORF or 5′UTR to enhance translation (Orom *et al.* 2008, Lee *et al.* 2009), the 3′ UTR has been the target for miRs in the clinic. The regulation of the miR expression by hormones and cytokines will be further detailed here with a focus on the tumor and tumor associated microenvironment cell types.

The clinical application of miRs has rapidly matured from aspirational to now exploiting its diagnostic and therapeutic potential. We refer the reader to the comprehensive reviews describing the mechanisms of miR action (Wagner *et al.* 2014, Fendler *et al.* 2016, Masliah-Planchon *et al.* 2016). The direct role of miRs in cancer was made by the seminal publication of mutations in miR processing enzymes, *DICER* and *DROSHA* leading to ovarian cancer (Merritt *et al.* 2008). Now there are two observational clinical trials for ovarian cancer patients to determine the effects on miR expression in patients (ClinicalTrials.gov identifier: Nbib1970696 and Nbib1572467). The pleotropic functions of miRs associated with transforming growth factor-beta/bone morphogenic protein (TGF-β/BMP) signaling will be used as an example as many of the miRs in current clinical utility seem to converge on this pervasive pathway. To appreciate the progress in the field, we need to start with one of the first mammalian miR identified, let-7. Its notoriety stems from KRas being one of its many targets. Although KRas is the most mutated gene in cancer, its activity is often elevated in the absence of mutations. Many miRs have been described to stimulate the RAS-MAPK pathway (miR-31, miR-143, miR-4689) (Hiraki *et al.* 2015, Chen *et al.* 2016, Edmonds *et al.* 2016). Regardless, in breast and ovarian cancer, let-7 is frequently downregulated, leading to increased KRas expression (Johnson *et al.* 2005, Dai *et al.* 2015). Since both of these cancers are commonly associated with activating Ras mutations, the loss of let-7 is even more significant in potentiating tumor progression. Although prostate cancer is not one of the cancers associated with Ras mutations through its tumor evolution, it is also associated with let-7 downregulation (Albino *et al.* 2016). The let-7 example points to one of the many similarities the three cancers have. However, the rationale for active clinical trials using let-7 mimetics has a broader basis, and that is the fact that let-7 is a potent down regulator of HMGB1, c-myc, IL-6 and cyclin D2. Thus, therapeutic targeting of a single miR can potentially address a multitude of genetic and epigenetic changes that need to be addressed when dealing with the heterogeneous disease of each ovarian, prostate or breast cancer patient. However, as with any ‘dirty drug’, antagonizing or mimicking a single miR with the many gene targets will result in unwanted side effects. Although the path from bench-to-bedside for miRs is not very different from any other oncology therapeutic or biomarker, the stark difference in miR mechanism-of-action make it important to understand this topic in the era of precision medicine. The mandate of the Precision Medicine Initiative (NIH) is to ultimately have data-driven care for patients. Cancer genomic and RNAseq analysis of patients is the most developed unbiased high throughput means of patient screening. Accordingly, miRs are prominent biomarkers currently being validated in the clinic today to support diagnosis, prognosis or therapeutic efficacy. Further, miRs are beginning to reach the clinic as therapeutics themselves to address some of the changes revealed by large-scale genomic analysis. However, it is important to remember miRs are generated by the cancer epithelia as well as its microenvironment. And in most cases, systemic administration of miR-based therapy will impact both cancer cells and its microenvironment. Thus, for miR studies to successfully translate to the patient, many cell types need to be considered (Sood *et al.* 2006).

Endocrine-related cancers of breast, prostate and ovaries will be the focus of this review. We discuss the mechanisms that regulate miRs during cancer progression. The clinical applications used to target miR functions increase precision, especially when standard therapies are less effective. Therefore, the urgency to understand the mechanisms by which miRs function during endocrine-related cancers is reflected in the studies focused on miR signaling in the tumor microenvironment.

## Rationale and pitfalls in targeting TGF-β/BMP signaling

**Figure 1:**
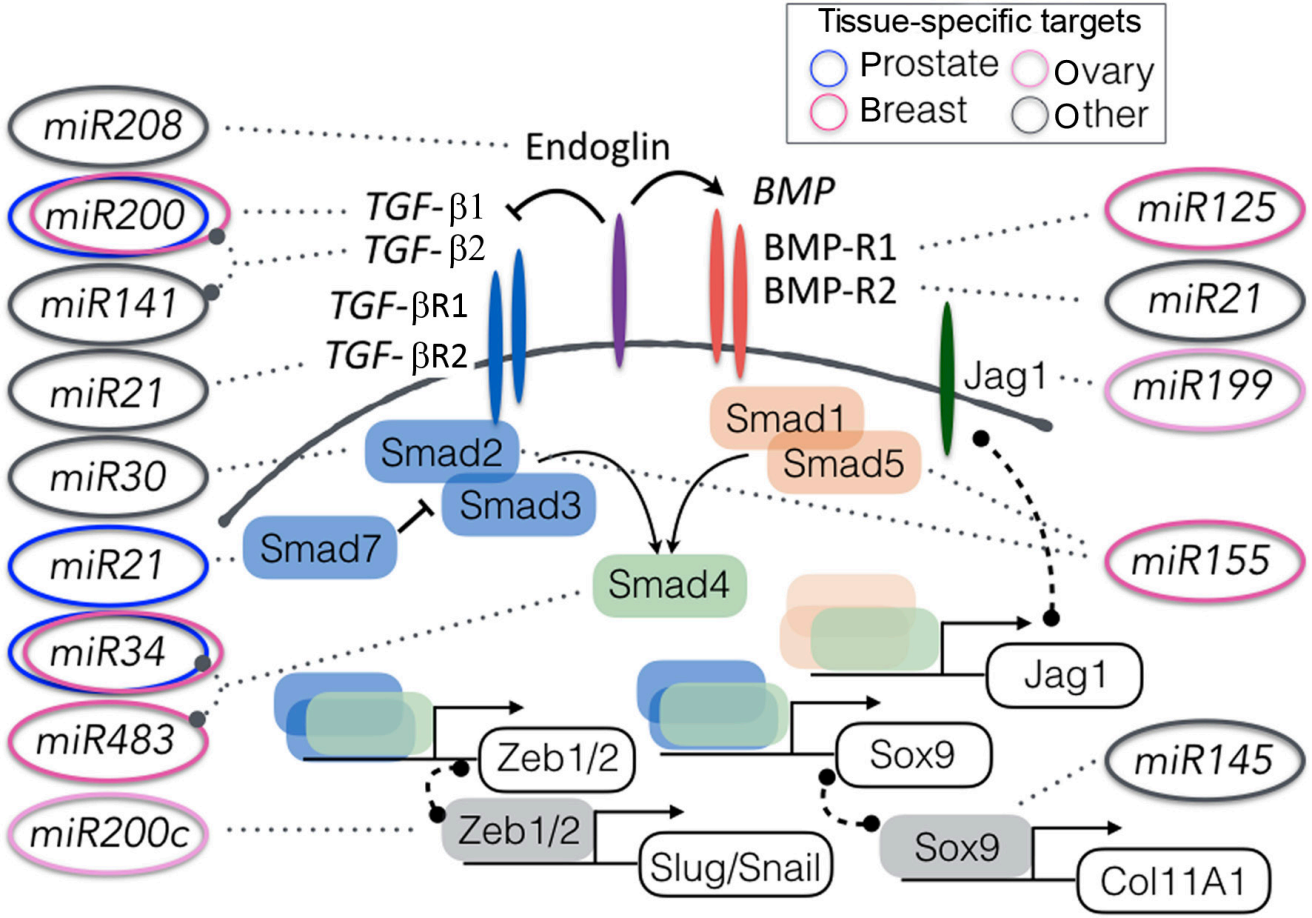
Modulation of the TGF-β/BMP signaling pathways by miRs. Validated targets in prostate, ovarian, and breast cancer are indicated by the corresponding colored ovals. miR targets identified in other tissues are indicated in grey ovals, as miR targets in one tissue may not be effective in another tissue. Only relevant miRs discussed in the paper are shown here and by no means depict all the TGF-β/BMP-associated miRs.

AS TGF-β signaling is a master regulator of tumorigenesis, the tumor microenvironment, and metastatic progression, this pathway impacts many miRs with significant clinical potential. The broad range of pathways influenced by TGF-β signaling is reflected in nearly 40 members comprising the TGF-β gene family. These members include the three TGF-β ligands, growth differentiation factors (GDFs), inhibin, activin, nodal, endoglin, lefty and BMP ligands (Chang *et al.* 2002). The complexities of stromal–epithelial interactions involving TGF-β signaling manifest itself as a tumor suppressor in tumorigenesis, yet demonstrate tumor promoting activity in metastatic progression (Bhowmick *et al.* 2003, 2004, Taylor *et al*. 2011, Lebrun 2012, Smith & Kang 2013, Neuzillet *et al*. 2015). The cross-talk between TGF-β signaling and miR functions is based on an autoregulatory feedback loop observed (Butz *et al.* 2011). The miR profiles of pituitary, prostate and breast cancer directly correlate with the disruptive functions of TGF-β. Several miRs, such as miR-21, miR-34a, and the miR-200 family cluster target TGF-β signaling in prostate, breast and thyroid cancer for their functions during tumor progression and promotion (Braun *et al*. 2010, Shen *et al*. 2012, Chen *et al.* 2016) (Fig. 1). Interestingly, miR-21 can also in turn promote TGF-β1-induced epithelial to mesenchymal transdifferentiation (Li *et al.* 2016). Conversely, miR-200 expression down regulates known targets, including TGF-β2, that are particularly up regulated in many cancers (Benlhabib *et al.* 2015). These few miRs serve just as examples of the cross-regulation with the expression with TGF-β ligands.

Small molecule and neutralizing antibody-based antagonism of the TGF-β signaling axis is being pursued for multiple cancers. However, with the pleotropic roles of TGF-β it was not surprising that targeting the TGF-β ligands or receptors was associated with some unwanted side effects (Kulkarni *et al.* 1993, Anderton *et al.* 2011). It is possible that the specificity of a miR to a particular TGF-β ligand isoform or downstream effectors may limit some of these unwanted effects. Of these effectors, Smad2, Smad3 and Smad4 activate downstream transcription, but can be inhibited by STRAP and Smad7 activation endogenously. In addition, p38MAP kinase, AKT and RhoA are downstream of TGF-β in a cell-specific manner, with distinct and overlapping functions with Smad signaling (Bakin *et al.* 2000, Bhowmick *et al.* 2001*a*,*b*, 2003). Smad2 and Smad3 are targeted by miR-155 and miR-200a, respectively (Kong *et al.* 2008, Park *et al.* 2008, Louafi *et al.* 2010, Ahn *et al.* 2012). Smad4, however, is targeted by miR-130a, miR-182, miR-205 and miR-483 (Hao *et al.* 2011, Geraldo *et al.* 2012, Egawa *et al.* 2016). Identification of miRs that target TGF-β downstream effectors presents an advantage of specificity. However, since Smad4 is a shared effector of much of the broader TGF-β family of ligands it may share some of the same side effects faced with the TGF-β receptor kinase inhibitors. The function of such miRs, if used in a therapeutic function, would ultimately be dictated by the pleotropic nature of the Smad proteins. For example, Smads regulate transcriptional repressors (i.e., Snail, ZEB1/2) associated with EMT, binding to proteins required to produce miRs (i.e., p68) and influencing microenvironment components, such as cancer-associated fibroblasts (CAF).

The role of TGF-β during the biogenesis and maturation of miRs has been analyzed extensively in several disease states. Both TGF-β and BMP signaling regulate vascular integrity, acting through the vascular smooth muscle cells. Inhibition of either TGF-β or BMP signaling can disrupt vascular smooth muscle cell contractile phenotype. TGF-β/BMP-induced miR-21 had an essential role in the expression of smooth muscle specific genes *CNN1* (calponin) and *ACTA2* (α-smooth muscle actin) (Davis *et al.* 2008). The study found that TGF-β/BMP signaling regulated miR-21 expression by potentiating Smad1/5 or Smad2/3 to p68, a component of the *DROSHA* miR processing complex (Davis *et al.* 2008). This indicated that TGF-β/BMP signaling can regulate miR biosynthesis and not just transcription. It could mean that many miRs, besides miR-21, may be regulated in this fashion. On its own, this does not necessarily affect miR-based therapeutic design, but it would suggest that miRs that target the Smad proteins might affect the biosynthesis of other miRs. Due to the broad target range of any individual miR, it would require that all such Smad-directed miR be tested for effects on other miRs.

## miRs impact epithelial–mesenchymal transdifferentiation

Epithelial–mesenchymal transition (EMT) is a multi-step process that regulates changes in cell morphology during embryogenesis, tissue development and tumor development (Kalluri & Weinberg 2009, Smith & Bhowmick 2016). One of the pro-tumorigenic roles of TGF-β signaling includes the potentiation of EMT (Oft *et al.* 1996, Bakin *et al.* 2000, Bhowmick *et al.* 2001*a*,*b*, 2003). The miRs associated with EMT have some overlap with those discussed regulated by TGF-β. They are important to note due to their relevance to tumor aggressiveness and therapeutic resistance. Snail1 and Slug are transcriptional repressors of epithelial markers (E-cadherin, zona occludin) and upregulate the expression of mesenchymal genes (vimentin, N-cadherin) (Thiery *et al.* 2009). miR-9 was found to downregulate Snail resulting in E-cadherin upregulation in melanoma to ultimately decrease growth and metastasis *in vivo* (Liu *et al.* 2012). Recently, miR-9 and miR-200c was found to regulate PDGFR-mediated endothelial differentiation of triple negative breast cancer (D’Ippolito *et al.* 2016). miR-9 inhibition or miR-200c restoration, delivered peritumorally to MDA-MB-231 xenografts, decreased the number of vascular lacunae, associated with breast tumor growth and dissemination (D’Ippolito *et al.* 2016). Another target of miR-200 is Zeb1/Zeb2 in the maintenance of cellular plasticity, a major hallmark of tumor cell morphology (Brabletz & Brabletz 2010). Interestingly, Zeb1 and Zeb2 bind to the E- and Z-box enhancer sequences of miR-200f promoter regions. In further cross-talk within the EMT regulators, Snail1 assists in the transcriptional repression of miR-200f, to enhance EMT (Diaz-Lopez *et al.* 2015). Importantly, miR-16 and miR-200 family members silences TGF-β signaling and blocks EMT (Brabletz & Brabletz 2010, Wang *et al.* 2014*b*, Tang *et al.* 2016). Individuals have the potential to influence several mRNA targets to regulate cancer progression (Davis-Dusenbery and Hata 2010). However, in the study of their endogenous expression in cancer, families of miRs can be expressed concomitantly to have broad effects. When inhibiting a miR, therapeutic efficacy may be limited as other miR family members can compensate for the targeted miR. However, the use of specific miR mimetics would not be plagued by this issue. In a specific example, ectopic expression of miR-200 members significantly reduced anaplastic thyroid carcinoma cell invasion in a ZEB1/ZEB2-dependent manner (Fig. 1) (Braun *et al*. 2010). As anaplastic thyroid carcinoma is a highly metastatic disease with poor prognosis, these findings suggest that a similar strategy for ovarian and triple negative breast cancer could be promising. It is important not to automatically assume that the regulation of a particular miR or its targets will be conserved from one tissue to another. However, findings in one tissue can suggest its testing in another tissue of interest. miR-10b is one such example where it was originally found to regulate EMT in a TGF-β dependent manner in breast cancer (Ma *et al.* 2007). Later, in independent mouse models, miR-10b was found to inhibit breast and pancreatic tumor metastasis (Moriarty *et al.* 2010, Ouyang *et al.* 2014). Although in both reports EMT was attributed to be rational for the role of miR-10b on metastatic potential, the targets identified were entirely different. Since the targets were not cross-validated in the two tissues, there is no way to know if the common phenotypic and physiologic readout of miR action was a result of the same target or targets. Despites the caveats on the specificity of miR targets, the role of miRs on EMT and metastatic progression are well supported.

## Addressing the tumor microenvironment with miRs

The concept of tumors being composed of disconnected ‘rogue’ masses of malignant cells has long been abandoned. We now understand that tumors function like organs, and the components that make up tumors and the surrounding environment plays intimate roles in cancer progression. The interactions between miR-21 and Smad7, for example, have been shown to predict the formation of reactive skin cancer-associated fibroblasts (CAF) (Li *et al.* 2013*b*). As a target of miR-21, Smad7 translation is inhibited leading to an increase in cells with the CAF phenotype. However, when miR-21 is blocked or Smad7 was overexpressed, TGF-β1-induced CAF formation was inhibited. The CAF and tumor epithelia of many cancer types rely on TGF-β signaling to mediate communication and increase tumor growth and potentiate therapy resistance (Coulouarn *et al*. 2008, Vonlaufen *et al*. 2008, Duner *et al*. 2010, Coulouarn & Clément 2014). However, due to the importance of the spatiotemporal nature of miR expression and action (Sood *et al.* 2006), we divided the discussion of specific miRs to the CAF associated with the three particular cancer types and immune cells in the next sub-sections.

### Regulation of miRs within the prostate tumor microenvironment

The prostate luminal and basal epithelia are encircled by smooth muscle cells that differentiate into activated fibroblastic cells during cancer progression. The tumor inductive nature of CAF was first coined in the context of prostate cancer (Olumi *et al.* 1999). CAFs are a major component of the tumor stroma with undoubted cancer-promoting effects. Of particular interest, TGF-β signaling influences CAF ability to mediate oncogenic transformation of normal epithelial cells (Bhowmick *et al*. 2004). The heterogeneous CAF populations can communicate within the stromal compartment in depositing certain ECM components and express cytokines/growth factors to facilitate cancer invasion and even instruct carcinoma cells to the site of metastatic invasion (Kiskowski *et al.* 2011, Li *et al.* 2013*b*). In prostate cancer, the CAF often lose TGF-β receptor type II expression due to promoter methylation (Banerjee *et al*. 2014). However, we have found that there is a juxtaposition of TGF-β-responsive and -nonresponsive CAF in the prostate cancer microenvironment (Kiskowski *et al.* 2011). The loss of TGF-β responsiveness in the CAF can lead to the accumulation of DNA damage and expression of multiple growth factors (Coppe *et al.* 2008, Banerjee *et al.* 2014). P53-induced miR-34a was found to restore DNA integrity (He *et al.* 2007, Yamakuchi *et al.* 2008). Interestingly, miR-34a expression in CAF has not been closely studied with respect to stromal DNA damage accumulation and its activation. Doldi and coworkers suggested that in prostate cancer the CAF could be activated in one of two ways: by IL-6 or TGF-β (Doldi *et al.* 2015). Both these cytokines can be sourced from the adjacent cancer epithelia *in vivo*. In this study, miR-133b emerged as a common mediator of CAF activation, as determined by the expression of markers such as, ACTA2, FAP, S100A4 and COL4A2 (Doldi *et al.* 2015). The expression of miR-409 by CAF was associated with prostatic tumor epithelial expansion and EMT (Josson *et al.* 2015). The surrounding prostate epithelia seemed to take up the miR-409, leading to increased tumor migration and invasion. Tumor growth continued due to miR-409-directed inhibition of RSU1 and STAG2 tumor suppressors. miR-15 and miR-16 are downregulated in prostate cancer CAF in the majority of 23 patients (Musumeci *et al.* 2011). These expression patterns inversely correlated with upregulation of FGF2 and FGFR1, supportive of the tumor inductive nature of CAF. In addition to these miRs that potentially serve as biomarkers for prostate cancer progression, Shen and coworkers found that plasma miR-21, miR-20a, miR-145 and miR-221 could risk stratify patients for tumor relapse (*n* = 82) (Shen *et al*. 2012). Patients with high or intermediate risk had particularly elevated expression of miR-21 (*P* = 0.047) and miR-145 (*P* = 0.011) compared to lower risk scores (Shen *et al*. 2012). Although the source of circulating miRs may be from either the cancer or the microenvironment, the capacity to affect the gene expression on cells that do not express the miR itself is eye-opening when it comes to expression profiling tumors, as is the practice of precision medicine. It also means that therapeutic targeting of miRs needs to consider both the tumor epithelia and its microenvironment.

### Regulation of miRs within the ovarian tumor microenvironment

All three major cell types of the ovaries (epithelial, germ and stromal cells) can develop malignancies. Although the epithelial tumors are the most common, the contribution of CAF in this disease is evident by shear bulk and prevalent cross-talk. The fibrotic scarring and development of fibroblast populations closely related to the epithelial tumor cells seem to correlate with miR functions. It has been difficult to stratify serous ovarian cancer. The pathologic similarities of this common ovarian cancer type do not reveal the level of chemoresistance of these tumors. However, a set of ECM and ECM remodeling proteins (10-gene signature: *AEBP1*, *COL11A1*, *COL5A1*, *COL6A2*, *LOX*, *POSTN*, *SNAI2*, *THBS2*, *TIMP3* and *VCAN*) were identified as associated with poor overall survival (Cheon *et al.* 2014). The signature genes are TGF-β regulated and their involvement with miR functions is broad. MMPs, TIMPs and Smads have a defined interplay with miRs in cancer metastasis. Examining miR silencing of Smad3 by miR-200a, for example, resulted in downregulation of a different set of ECM proteins (i.e., collagen I, collagen IV, fibronectin, PAI-1 and α-smooth muscle actin) (Wang *et al.* 2011). Careful interrogation of the ovarian cancer-associated signature resolved *COL11A1* to be a determinant of lethal disease progression (Cheon *et al.* 2014). *COL11A1*expression is regulated by a *TGFβ* target transcription factor, *SOX9*. SOX9 is a direct target of miR-145 and is found to regulate *COL11A1* expression (Yang *et al.* 2011). As the primary tumor microenvironment can predispose the cancer epithelia for metastasis, the metastatic site itself clearly has an influence on the adaptability of the tumor cells (Bhowmick 2012). One way the secondary site can be made adaptive to the tumor cells is the transport of cancer-derived miRs via exosomes. A seminal example of this phenomenon was described for pancreatic cancer metastasis to the liver (Hoshino *et al.* 2015). As another highly metastatic tumor type, ovarian cancer frequently spreads to the omentum adipose fat. In one example, the omental fat cells and associated CAF were found to express exosomes containing miR-21 (Au Yeung *et al.* 2016). miR-21 is induced by many cytokines including TGF-β and is associated with collective migration (Dean *et al.* 2015). The ovarian cancer epithelia were found to accept these miR-21 containing exosomes to support chemoresistance by directly silencing its target, APAF1 (Au Yeung *et al.* 2016). Metastatic breast and prostate cancer cells also overexpress miR-21. miRs have been identified in exosomes and microvesicles derived from several tissues, plasma, saliva and urine to support a means of cell to cell transfer and the viability of the use of exosomes as biomarker capsules. Thus, it is fitting that therapeutic microvesicles can be generated to transfer miR cargo to counter metastatic progression.

### Regulation of miRs within the breast tumor microenvironment

miRs identified in CAF can regulate the expression of paracrine factors. Dysregulated miRs in breast CAF included upregulation of miR-221, miR-31, and miR-221 with the downregulation of miR205, miR-200b, miR-200c, miR-141, miR-101, miR-342, let-7g and miR-26b affecting all aspects of cell differentiation and paracrine regulation (Zhao *et al.* 2012). Although in the epithelia miR-205 and miR-200 family members (miR-200c, miR-200b and miR-141) are associated with EMT progression, in fibroblastic cells they clearly have a different function. In the breast CAF, miR-200 family members seem to contributed to ECM remodeling through the expression of fibronectin and lysyl oxidase to potentiate cancer epithelial invasion (Tang *et al.* 2016). In the absence of somatic mutations in the CAF population, epigenetic changes in the form of DNA methylation and miR expression can dictate the fate of adjacent epithelia. However, the redundancy of miR-200 downregulation in both the epithelial and stromal compartments is highly supportive of using miR mimetic of this species.

We have discussed examples where the miR itself can act in a paracrine manner. Another example of this for breast cancer was identified in exosomes expressed by CAF recently. Upregulation of miR-133b in CAF was found to travel via gap junction proteins into breast cancer epithelia, where it has anti-apoptotic and pro-growth molecule with oncomiRs properties (Katakowski *et al.* 2010, Xin *et al.* 2012). Interestingly, miR-133b transfer could be limited by the use a gap junction inhibitor (Xin *et al.* 2012), revealing a novel way of countering the miRs identified in primary tumors to prevent future metastatic progression. Incidentally, miR-133b was also identified to be a poor prognostic indicator in prostate cancer, in causing further aggressive behavior (Li *et al.* 2014). For prostate cancer, miR-133b was of epithelial origin. But the same miR-133b expressed by breast cancer CAF suggests that an individual miR can have a role in the cancer and its microenvironment regardless of its source.

### miR regulation in immune system signaling

The introduction of checkpoint inhibitors in the armamentarium against cancer has caused us to reevaluate our understanding of T cell biology, in terms of clinical applications. Now, companion biomarkers and combination therapy candidates are a focus in many labs. miRs can play an important role in both these fronts. As TGF-β is a known immune-suppressor, it is not surprising that it has a central role in adaptive and innate immune function. The Smad2 silencing by miR-155 can promote B-cell activation as well as enable CD4 T cells mature to T_H_17 and T_H_1 cells (Mehta & Baltimore 2016). miR-155 is also critical in cells of the myeloid lineage. miR-155 expression is upregulated by NF-κB signaling, downstream of toll-like receptor activity to drive dendritic cells and macrophage differentiation (O’Connell *et al.* 2007). Smad4-mediated stimulation of granulocyte differentiation by TGF-β is tempered by miR-130a expression (Hager *et al.* 2011). The miR-34a is a member of miR-34 family that target TGF-β/Smad4 signaling in T-regulatory (Treg) cell tumor recruitment. Yang and coworkers demonstrated that miR-34a downregulation in liver tissues results in CCL22 expression for the recruitment of Treg-mediated escape from immune surveillance (Yang *et al.* 2012). Numerous publications on miR-34a suggesting its role as a tumor suppressor, demonstrate a reduction in several cancer types including breast, ovarian and prostate cancer (Corney *et al.* 2010, Peurala *et al.* 2011, Benassi *et al.* 2012). Analysis of miR-34 in human epithelial ovarian cancer showed that there was a 100% decrease in miR-34, and a 72% decrease in miR-34b*/c in the context of p53 mutation (Corney *et al*. 2010). Wild type p53 correlated with a 93% decrease in miR-34 in ovarian cancer cells. Clinically, stage III and stage IV tumors were analyzed where reduced miR-34 was significant (*P* = 0.0029, *P* = 0.0171, respectively). Interestingly, miR-34a downregulation is frequently regulated by promoter methylation in cancer cells. These counter acting epigenetic regulations impact the expression of endogenous oncogenes (e.g., MYC) as well as exogenous immune infiltration (Benassi *et al.* 2012, Yang *et al.* 2012). The confounding epigenetic mechanisms need to be considered before long-term clinical use of miR-based therapeutics is in practice.

## Clinical applications of miRs in ovarian, prostate and breast cancer

miRs are currently part of many trials examining genomic signatures to contend with the issue of patient selection to identify companion biomarkers for conventional therapeutics. As miRs are increasingly examined in patients, the importance of not making generalizations of a miR being oncogenic or tumor suppressor is clear. Further sources of variability in miR detection in bodily fluids can be associated with factors such as diet, age and circadian rhythms. Yet, in multiple independent studies miR-200 family was upregulated in metastatic breast and prostate cancers (Lin *et al.* 2014, Taylor *et al.* 2016). However, miR-200 family members have been reported to be both up- as well as downregulated in ovarian cancer (Park *et al.* 2008, Kan *et al.* 2012). Notwithstanding, miRs are attractive due to their stability and conservation among species (Chen *et al.* 2008). miRs gain their extraordinary stability from their small size (20–24 nt) being less exposed to degradation (even during FFPE sample processing), encapsulated in protective exosomes or microvessicles and most commonly its association with RNA binding proteins. Exosome-free miRs associated with argonaute proteins make up much of what is detectable in circulation (Turchinovich *et al.* 2011). It is not just a coincidence that miRs have diagnostic value for cancer patients, but rather that miRs are frequently located at fragile chromosomal sites associated with cancer hotspots (Calin & Croce 2006). Accordingly, screening both free and exosome-associated miRs by high-throughput sequencing platforms have revealed changes in breast, prostate and ovarian cancer patients.

RNA-based therapies have been tested for twenty years, with antisense oligonucleotides having the longest track record in the clinic. As of 2016, eight of those therapies had reached phase III clinical trials. Short interfering RNAs (siRNAs) have been used in the clinic, but none have reached phase III yet. However, there are only two current trials using miRs in interventional trials. In these phase I studies, miR mimetics have been used to restore miR-34 (MRX34) and miR-16 (TargomiRs) activity. Some of the roles of miR-34 have already been discussed, as one of its targets is Smad4. As previously described, miR-16 inhibits TGF-β-induced EMT by silencing p-FAK and p-Akt expression, disrupting NF-κB and Slug transcriptional activity (Wang *et al.* 2014*b*). Although it is not possible to assess the clinical efficacy of the miR-based therapeutics at this stage, these two drugs were well tolerated with minimal inflammatory side effects. Regardless, it should be noted that the FDA has not approved an RNA-based drug to date. The reasons for the low success rate are not entirely clear, but a critical hurdle of therapeutic delivery seems to have improved. The lessons learned through the long history of antisense RNA-therapy are evident, as both miR trials use nanoparticle-based formulations to enhance drug availability. In the near term, we may also see further improvements in tissue targeting and sustained release of miRs or anti-miRs encapsulated in nanoparticles conjugated to antibodies or peptides. However, with about a 7% likelihood of FDA approval of any oncology drug, many of the challenges are not unique to RNA-based therapy (Hay *et al.* 2014). The need for understanding tumor heterogeneity, mechanisms of host–tumor interaction, and patient selection are only a few obvious issues that we still need to address.

### miRs in prostate cancer patient care

Prostate cancer is the most common non-cutaneous cancer in men and, next to lung cancer, is the second leading cancer killer in men. Improvements in survival from the disease have been due to advances in first line interventions (surgery and radiation) as well as newer antagonists to the androgen signaling axis. Yet, metastatic prostate cancer has no cure as resistance to androgen antagonists are inevitable, and taxane therapy was approved by the FDA for providing an increase in three months of overall survival (Petrylak *et al.* 2004, Tannock *et al.* 2004). Thus, markers for patient selection and sensitizing agents for current therapies are a focus in the field. Currently, there are a number of clinical trials that aim to analyze the miRs in prostate cancer and treat the malignancies by using miRs, detailed in Table 1. As an example, the Medical College of Wisconsin conducted a study to identify exosomal miRs from peripheral blood of prostate cancer patients with systemic disease to predict response to androgen deprivation therapy. In the study, they gathered blood sample at the time of treatment, 3 months post-treatment and upon disease progression to determine changes on disease response (ClinicalTrials.gov Identifier: Nbib2366494). The study further aims to compare exosomal RNA levels between groups of patients who have relapsed as opposed to those in remission within 2 years of treatment. In a phase II study, expression levels of circulating miRs are being validated as a biomarker for sensitivity to abiraterone acetate (androgen synthesis inhibitor) and to predict metastatic prostate cancer progression (ClinicalTrials.gov identifier: Nbib1503229). These observational and interventional study examples would help in the process of decision-making for patient care. One could envision such a set of markers complementing imaging techniques in identifying metastatic status. Most prostatic metastasis is invisible to current imaging techniques in the early stages. If stable miRs could predict such an eventuality, it could suggest the use of chemotherapy, such as a taxane, at a time when it could appreciably alter overall survival.

**Table 1:** Prostate cancer clinical trials involving miRs.

Trial reference	Study type	Institution	Trial title
NCT2366494	Observational	Medical College of Wisconsin	MicoRNAs to predict response to androgen deprivation therapy
NCT1503229	Interventional	University of Washington	Abiraterone acetate in treating patients with metastatic hormone-resistant prostate cancer
NCT1220427	Observational	Wuerzburg University Hospital	microRNA expression profiles in high risk prostate cancer
NCT2471469	Observational	Radboud University	Personalizing enzalutamide therapy by understanding the relation between tumor mRNAs, miRNAs and treatment response
NCT2391051	Interventional	University of Erlangen-Nürnberg Medical School	Focal brachytherapy in patients with selected ‘low-risk’ prostate cancer – a phase-II-trial

Another set of observational trials attempts to identify therapeutic efficacy by measuring dynamic changes in the expression of specific miRs in circulation in response to androgen deprivation therapy. A current study underway at Radboud University (Nijmegen, Netherlands) explores the possibility of dosing enzalutamide therapy (androgen receptor antagonist) based on reduction in a panel of select miRs to assess treatment response (ClinicalTrials.gov identifier: Nbib2471469). A phase II trial conducted by University of Erlangen-Nürnberg Medical School (Nürnberg, Germany) explores the feasibility and toxicity of focal brachytherapy in 50 patients with low-risk prostate cancer (ClinicalTrials.gov identifier: Nbib2391051). The secondary outcome of this study is to correlate miR-375 and miR-141 to visible efficacy of the radiation therapy. This secondary endpoint is supported by a number of previous clinical studies including, one where miR-21, miR-141 and miR-221 was detected in the plasma of a prostate cancer cohort of 51 patients (locally advanced or metastatic) had higher expression compared to 20 healthy controls (Yaman Agaoglu *et al.* 2011). It turned out that miR-21 helped distinguish between healthy and prostate cancer patients, but miR-141 (miR-200 family member) enabled distinction between localized and metastatic subjects. A related study screening 667 abundant miRs in the serum of prostate cancer patients identified miR-375 and miR-141 to be closely associated with disease progression (ClinicalTrials.gov identifier: Nbib2391051) (Brase *et al.* 2011). The study therefore proposed that circulating miR-375 and miR-141 can be used as a non-invasive biomarker for tumor progression (Brase *et al.* 2011). The near-term ramifications of determining treatment efficacy is to be able to change therapies to one that may be effective for the patient and not subject him to the side effects associated with an ineffective drug. However, it may also suggest an opportunity to provide a combination therapy of a particular miR mimetic with a conventional intervention. For those patients that do not manifest a drop in miR-141 with radiation, an anti-miR-141 could be supplemented with radiation. As the use of miRs for prostate cancer therapeutics is at a nascent stage, results from such observational trials could lead back to the bench to test potential complementary therapeutic approaches pre-clinically.

The first-in-man miR-based therapeutic trial, MXR34 (Nbib1829971), delivered in a liposomal nanoparticles was in part based on the important work by Liu and coworkers showing that systematic delivery of miR-34a reduced prostate tumor and metastasis in mice (Table 1) (Liu *et al.* 2011). As CD44 was validated as a target for miR-34a in the publication, it was attributed to preventing prostate cancer metastasis and regeneration (Liu *et al.* 2011). *CD44* silencing in LAPC4 cells prevented lung metastasis and expansion of orthotopic tumors (Liu *et al.* 2011). Since that study, we have a better appreciation of the pro-metastatic properties of hyaluranon (a CD44 ligand) (Hiraga *et al.* 2013). However, considering other known targets of miR-34a, such as Notch, CDK4 and CyclinD1, it is difficult to attribute a miR’s systemic effects *in vivo* on any one target. As a direct target of p53, miR-34 targeting can potentially have effects on any number of cancer types. But, the highest levels of miR-34a are in the ovary, prostate and testes. Thus, its downregulation in the respective cancers in those organs would likely benefit the greatest from a miR-34 mimetic like MXR34. However, as with all miRs, the targets are highly dependent on the cell type. For the tested prostate cells, CD44 has emerged as the reproducible target of miR-34a. However, it is important to note that the current MXR34 study in unresettable liver cancer patients where delivery system and potential off-target effects will be revealed. Liver cancer was the chosen disease for the MRX34 study since the liposomes that contain the double-stranded RNA payload tend to accumulate in liver tissues. For prostate targeting, the lyposomes maybe functionalized with PSMA (prostate specific membrane antigen) or other such agent. MXR34 tries to address the two primary obstacles in gene therapy, tissue-specific delivery and cellular uptake.

### miRs in ovarian cancer patient care

The most lethal form of gynecologic malignancy is ovarian cancer, with a dismal 44% five-year survival rate. Over 200,000 women around the world are diagnosed with ovarian cancer each year and cancer antigen 125 (CA-125) can only detect late stage disease with little predictive value as to therapy response or relapse. Unfortunately, since three-fourths of the patients present with advanced disease, surgical cytoreduction and platinum/taxane-based chemotherapy are among the first lines of intervention. Benson and coworkers first reported that the concentration of circulating miRs is altered after administration of carboplatin and decitabine chemotherapy regime in platinum resistant recurrent ovarian cancer patients, in a phase II clinical trial of 18 subjects (Benson *et al.* 2015). A tumor suppressive role for miR-148b-5p has been suggested in multiple solid tumors (Song *et al.* 2011, Zhao *et al.* 2013). The phase II trial showed reduced circulating miR-148b-5p was associated with longer progression free survival on chemotherapy (Benson *et al.* 2015). This study suggested that chemotherapy induced miRs concentration changes could be used as a prognostic biomarker of therapeutic response in ovarian cancer patients. A similar decision enabling miR was described for prostate cancer. However, for a disease that can progress rapidly, miR-448-5p could be especially important to limit ovarian cancer recurrence (Kim *et al.* 2010).

EphA2 is a well-recognized marker and evolving therapeutic target for multiple cancers including ovarian and breast cancer. EphA2 is part of a receptor family associated with epithelial–endothelial interaction via juxtacrine signaling by Eph ligands. Interestingly, miR-520d-3p with EphA2 is an independent prognostic marker for serous ovarian cancer (Nishimura *et al.* 2013). Many mechanisms of EphA2 targeting involving peptide- and antibody-conjugates of chemotherapy as well as CAR-T cell therapy have had preclinical promise (Kiewlich *et al.* 2006, Chow *et al.* 2013, Wang *et al.* 2013). SiRNA-mediated silencing of EphA2 has reduced ovarian tumor growth in mouse models (Landen *et al.* 2005). More recently, miR-520d-3p mimetic was found to synergize with EphA2 siRNA to reduce ovarian tumor size and invasive capacity (Nishimura *et al.* 2013). Both complementary therapeutic platforms depend on AGO2-RISC complex to downregulate EphA2 expression.

TargomiRs is the second of the two miR mimetics currently in clinical trials (Australian New Zealand Trial # ACTRN12614001248651). It is being tested for safety in late stage pleural mesothelioma and non-small cell lung cancer subjects. TargomiRs are EGF receptor-targeted antibody conjugated to minicells containing a miR-16 mimic. The mimic is a double-stranded RNA molecule in a bacteria-based nanoparticle (minicell). As previously mentioned, miR-16 is implicated as a tumor suppressor for many cancers. However, there are compelling preclinical reports in which the ovarian cancer models were treated with cisplatin alone or in combination with miR-15a and miR-16 mimetics (Dwivedi *et al.* 2016). There was a significant inhibition of tumor growth of the chemo-resistant ovarian cancer cells by the addition of the miR mimetics that targeted BMI1 and the cisplatin transporter, ATP7B (Dwivedi *et al.* 2016). The study demonstrated that *in vivo* nano-liposomal delivery of miR-15a and miR-16 decrease tumor growth in preclinical chemo-resistant orthotopic ovarian cancer mouse model in support of combination therapies. In another study, restoration of miR-199b-5p increased sensitivity to cisplatin induced cytotoxicity *in vitro* and *in vivo* ovarian cancer models via inactivation of Jag1 mediated Notch signaling pathway (Liu *et al.* 2014*b*). A combination therapy possibility with miRs and taxane-based chemotherapy was also suggested. With the reports miR-200c role in ZEB1/ZEB2-mediated EMT progression, Cittelly and coworkers found that restoration of miR-200c induced chemo- and ankoikis sensitivity (Cittelly *et al.* 2012). Furthermore, miR-220c restoration also helped reduce tumor growth alone and in combination with paclitaxel (Cittelly *et al.* 2012). The point of this study was to test if the recovery of miR-200c can improve response to chemotherapy. Ovarian cancer has a high rate of relapse within 18 months of current therapeutic interventions with no targeted therapies approved. To identify patients that will benefit from current lines of chemotherapy, an interventional trial profiling serum miRs from patients prior to chemotherapy treatment is being performed (ClinicalTrials.gov identifier: Nbib1391351, Table 2). For those that may not fare as well on chemotherapy, miRs may be used to overcome drug resistant variants that emerge in ovarian cancer progression.

**Table 2:** Ovarian cancer clinical trials involving miRs.

Trial reference	Study type	Sponsor	Trial title
NCT2758652	Observational	Tampere University Hospital	Molecular mechanisms leading to chemo-resistance in epithelial ovarian cancer
NCT1391351	Interventional	Centre Francois Baclesse	Search for predictors of therapeutic response in patients with carcinoma of the ovary, the fallopian tube or peritoneal serous-type advanced
NCT1572467	Observational	Children’s Oncology Group	DICER1 mutations and miRNA in ovarian and testicular sex cord stromal tumors of childhood
NCT1970696	Observational	Children’s Hospitals and Clinics of Minnesota	International ovarian and testicular stromal tumor registry
NCT1879436	Observational	Meir Medical Center	The effect of human placental explants and pregnant women sera on cancer cells

### miRs in breast cancer patient care

Breast cancer is the second leading killer of women, with only lung cancer resulting in more cancer deaths. Yet, more than 3.1 million women with a history of breast cancer are alive in the US with the potential need for further means of intervention. The estrogen receptor positive subtype is a critical discriminator, accounting for nearly 75% of all cases of breast cancer. However, non-hormone responsive (triple negative breast cancer) results account for the poor prognosis, often treated with a cytotoxic chemotherapy or/and targeted therapy (with exception of HER2 antagonists). As with prostate cancer, hormone responsive breast cancer can evolve to a hormone-nonresponsive disease in heterogeneous tumors. As with other cancers discussed, there is a lack of reliable biomarkers for patient selection and therapeutic efficacy where miRs can play an important role. Heneghan and coworkers demonstrated that among the elevated breast cancer-associated miRs present in circulation, miR-195 can help distinguish from other malignancies (melanoma, renal, prostate and colon cancer) and from non-malignant subjects (Heneghan *et al.* 2010). All the cancer patients generally expressed let-7a, miR-10b and miR-155, but miR-195 provided strong specificity and sensitivity with other covariates.

With MXR34 being tested and the breadth of supporting literature for its application, many patients including those with breast cancer may benefit following pharmacokinetics/maximum tolerated dose (PK/MTD) are established in the phase I trial. In a recent report, anti-tumorigenic effect of miR-34a administration in triple negative breast cancer models inhibited proliferation, invasion and metastasis (Adams *et al.* 2016). Moreover, the delivery of miR-34a replacement therapy retarded the tumor growth of subcutaneously and orthotopically xenografted tumors (Adams *et al.* 2016). In a seminal publication where breast cancer patients were treated with the chemotherapeutic 5-flurouracil (5-FU), miR-34a was found to be downregulated (Li *et al.* 2013*a*). Accordingly, a miR-34a mimetic was given with chemotherapeutic 5-flurouracil (5-FU) to reveal a more efficient anti-tumor effect than either single agent treatment (Li *et al.* 2013*a*). These studies support the promise of miR-34a (MXR34) as a potential therapeutic agent for breast and other cancer patients.

Majority of the breast cancer clinical trials are observational in terms of profiling circulating miR expression, like the one by Cancer Trials Ireland which aims to identify miR in response to neoadjuvant and adjuvant chemotherapy (ClinicalTrials.gov identifier: Nbib1722851, Table 3). Similarly, another clinical study investigates the circulating miRs as markers of hormone resistance and sensitivity in patients with metastatic invasive breast cancer or locally advanced breast cancer (ClinicalTrials.gov identifier: Nbib1612871, Table 3). It is worth noting the paradigm set by identifying miRs as a surrogate of side effect of certain chemotherapies. Doxorubicin (Adriamycin, Rubex), used for many cancers including breast cancer, is a DNA damaging agent that can cause cardiac damage. Years of work on miRs in the cardiology field has revealed miR-208 to be elevated in cases of cardiac hypertrophy and heart failure (Callis *et al.* 2009, Montgomery *et al.* 2011, Oliveira-Carvalho & Bocchi 2016). miR-208 expression is associated with fibrosis and EMT progression as well as specifically targeting the BMP co-receptor, endoglin (Fig. 1) (Liu *et al.* 2014*a*, Wang *et al.* 2014*a*). If the clinical trial demonstrates miR-208 as a robust marker for cardiac damage, there will be a pre-clinical basis to consider the use of an antimiR-208 as a viable cardio-protective for chemotherapy patients (Callis *et al.* 2009, Montgomery *et al.* 2011, Tony *et al.* 2015). Such an application of a miR-based therapy would follow the logic of a preclinical study where Xue and coworkers identified miR-621 elevation associated with paclitaxel and carboplatin (PTX/CBP) sensitivity (Xue *et al.* 2016). They subsequently administered miR-621 mimetic to silence the FBXO11, a poor prognostic indicator, in sensitizing breast tumors to PTX/CBP (Xue *et al.* 2016). As most of the miR-associated trials listed in Tables 1, 2 and 3 are observational, in identifying changes in miR expression, this paradigm of targeting the deregulated miRs for therapy could follow. With appropriate pre-clinical validation, these same miRs may be targeted by a miR mimetic or anti-miR. However, as with any such line of reasoning, not all good biomarkers are consequential to disease etiology and conversely, miRs that are central to disease progression or therapeutic resistance may not serve to be robust biomarkers.

**Table 3:** Breast cancer clinical trials involving miRs.

Trial reference	Study type	Sponsor	Trial title
NCT1722851	Observational	Cancer Trials Ireland	Circulating miRNAs: novel breast cancer biomarkers and their use for guiding and monitoring response to chemotherapy
NCT2127073	Interventional	Sheldon Feldman	Pilot study of oxytocin and microRNA identification in NAF, serum, and tissue in women with breast cancer
NCT1957332	Observational	University Medical Center Groningen	Imaging patients for cancer drug selection – metastatic breast cancer (IMPACT-MBC)
NCT1598285	Observational	Spanish Breast Cancer Research Group	A combined GWAS and miRNA for the Identification of bevacizumab response predictors in metastatic breast cancer
NCT1231386	Observational	City of Hope Medical Center	miRNAs profiling of breast cancer in patients undergoing neoadjuvant or adjuvant treatment for locally advanced and inflammatory breast cancer
NCT1612871	Interventional	Institut Claudius Regaud	Circulating miRNAs as biomarkers of hormone sensitivity in breast cancer? Pilot study
NCT2656589	Observational	Sun Yat-Sen Memorial Hospital of Sun Yat-Sen University	A perspective study of the predictive value of microRNA in patients with HER2 positive advanced stage breast cancer who were treated with herceptin
NCT2065908	Observational	West Pomeranian Cancer Center	Circulating microRNAs as a novel biomarker of early cardiotoxicity in breast cancer patients treated with anthracyclines
NCT581750	Observational	Memorial Sloan Kettering Cancer Center	Molecular genetic basis of invasive breast cancer risk associated with lobular carcinoma *in situ*
NCT1965522	Interventional	Juravinski Cancer Center	Anti-proliferative effects of vitamin D and melatonin in breast cancer (MELO-D)
NCT2288806	Interventional	Hamilton Health Sciences Corporation	Melatonin and vitamin d in breast cancer (MELO-D)
NCT2103140	Interventional	Georgetown University	An exercise randomized controlled trial targeting African-American women with metabolic syndrome and high risk for breast cancer
NCT1907438	Observational	Hadassah Medical Organization	Identification of the transformation potential of normal estrogen exposed BRCA1 (breast cancer susceptibility gene 1) and BRCA2 (breast cancer susceptibility gene 2) heterozygous epithelial breast cells due to irradiation
NCT773695	Interventional	Hoffmann-La Roche	A multicenter, randomized, ph II clinical trial to evaluate the effect of avastin in combination with neoadj treatment regimens on the molecular and metabolic characteristics and changes in the primary tumors with ref to the obtained responses in patients with large primary HER2 Neg breast cancers
NCT1724450	Interventional	University of Sao Paulo	Carvedilol effect in preventing chemotherapy – induced cardiotoxicity. a randomized double blind study
NCT2437318	Interventional	Novartis Pharmaceuticals	A phase III randomized double-blind, placebo controlled study of alpelisib in combination with fulvestrant for men and postmenopausal women with hormone receptor positive, HER2-negative advanced breast cancer which progressed on or after aromatase inhibitor treatment
NCT1879436	Observational	Meir Medical Center	The effect of human placental explants and pregnant women sera on cancer cells
NCT2678650	Interventional	Capital Medical University	MicroRNA mediates volatile anesthetics preconditioning induced artery protection

## Summary and perspective

Many examples of miR-based biomarkers and potential therapeutics in prostate, ovarian and breast cancers have been presented. These examples serve to highlight the mechanism of action and ongoing clinical trials for the diseases. In the post-genomic age a trove of patient/tumor-specific data can potentially guide and prioritize the clinical findings. However, the challenge in the field is the ability to predict miR target sites with high confidence in a cell type of interest. miRs typically repress target gene expression, but the reciprocal effect on targets by miRs is less clear. Although miR-based therapeutics is in its infancy it faces many of the same limitations as other targeted therapies. As the miR field progresses the focus may be less on a specific target, but a genomically defined phenotype. Conceptually, miRs could complement CRISPR/Cas9 in engineering networks. As we better characterize the heterogeneous nature of a patient’s tumors and consider genetic/epigenetic drift associated with disease progression and therapeutic response, miR-based therapies become more attractive due to their stability, sequence specificity, and relative ease of synthesis.

## Declaration of interest

The authors declare that there is no conflict of interest that could be perceived as prejudicing the impartiality of this review.

## Funding

This work was supported by the Veterans Administration Merit Award (grant number bib1BX001040) and National Cancer Institute at the National Institutes of Health (grant number R01CA108646).
